# Image guided focused ultrasound: development of a comprehensive treatment planning, monitoring and control, and assessment

**DOI:** 10.1186/2050-5736-3-S1-P10

**Published:** 2015-06-30

**Authors:** Costas Arvanitis, Gregory Clement, Nathan McDannold

**Affiliations:** 1 Brigham & Women’s Hospital/Harvard Medical School, Boston, Massachusetts, United States; 2 Cleveland Clinic, Cleveland, Ohio, United States

## Background/introduction

MRgFUS offers unambiguous advantages over other current treatment modalities, as it is noninvasive and does not use ionizing radiation. Further, ultrasonically-controlled stable and inertial microbubble oscillations exert forces that can, among others, activate cell’s mechanoreceptors, disrupt cellular and vascular membranes, accelerate the dissolution of blood clots, enhance thermal ablation, and induce localized tissue erosionetc. FUS can also be used transcranially for tumor ablation, blood-brain barrier disruption, neuromodulation, etc. Harnessing and, potentially, combining these abilities holds great promise for therapy and diagnosis of cancer and cardiovascular and central nervous system diseases and disorders. For the wide-spread use of these approaches, development of methods and technology to enable precise planning, monitoring and control, and assessment of their outcome are essential.

## Methods

**Figure 1:**
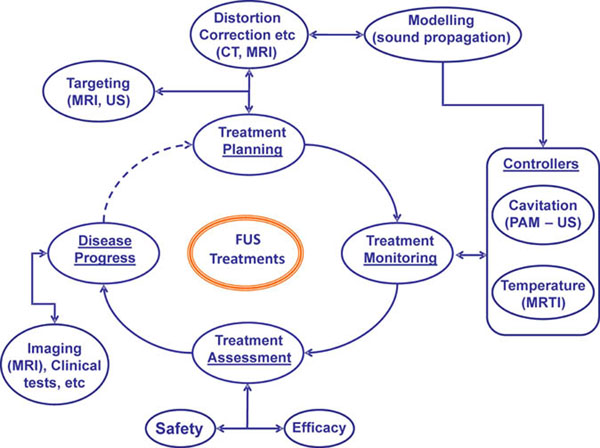
Image Guided FUS framework

We developed a novel noninvasive and clinically relevant framework (Fig. 1) that combines i) multimodality imaging (US/MR/CT), ii) 3D Finite Difference Time Domain (FDTD) numerical simulations, and iii) an integrated MR and US imaging FUS system for guiding FUS therapy. The proposed framework enables accurate treatment planning, via visualization of anatomic structures (MRI), extraction of their acoustic properties (CT) and simulation of US propagation (3D FDTD), which includes realistic microbubble acoustic emissions. The co-registration of CT (skull) with the MR and US datasets allowed us to model and study the microbubble emissions propagation through the skull and refine a standard PAM back-projection algorithm to account for spatially varying wave propagation effects, such as refraction and diffraction. The key component of our framework is the MR and US imaging guided FUS system that is used to monitor and control both the thermal and mechanical effects of FUS transcranially, here using manual and simple on-off controllers.

Finally, using MR contrast agent as drug surrogate, the proposed framework is used to assess drug uptake after FUS-BBB disruption.

## Results and conclusions

The incorporation of a variable speed of sound to the PAM back-projection algorithm corrected the aberrations introduced by the skull (NHP and Human). Further, more than 94% agreement in the FWHM of the axial and transverse line profiles between the simulations incorporating microbubble emissions and experimentally-determined PAMs was observed in NHP. The acoustic emissions monitoring allowed to fine-tune the sonication power for efficacious FUS-BBB disruption in glioma-bearing rat, while, MR temperature imaging based FUS controller allowed to attain constant focal temperature (temperature rise: 7±1.5 0C, for 5 mins) in the brain of a healthy rat. These abilities that have not been previously shown provide a clinically relevant framework for guiding FUS in the brain. We envision that the proposed framework will be essential for developing, optimizing, and translating current and new therapeutic FUS approaches to the clinics.

